# Choosing a maternity hospital: a matter of travel distance or quality of care?

**DOI:** 10.1007/s43999-024-00041-1

**Published:** 2024-05-09

**Authors:** Daniela Koller, Werner Maier, Nicholas Lack, Eva Grill, Ralf Strobl

**Affiliations:** 1 Institute of Medical Data Processing, Biometrics and Epidemiology (IBE), Faculty of Medicine, Marchioninistr. 15, 81377 Munich, Germany; 2 Bavarian Institute for Quality Assurance, Munich, Germany; 3 German Center for Vertigo and Balance Disorders, University Hospital, LMU Munich, Marchioninistrasse 15, 81377 Munich, Germany

**Keywords:** Secondary data, Hospital choice, Quality, Birth, GIS, Deprivation, Access, Health policy

## Abstract

**Background:**

The choice of a hospital should be based on individual need and accessibility. For maternity hospitals, this includes known or expected risk factors, the geographic accessibility and level of care provided by the hospital. This study aims to identify factors influencing hospital choice with the aim to analyze if and how many deliveries are conducted in a risk-appropriate and accessible setting in Bavaria, Germany.

**Methods:**

This is a cross-sectional secondary data analysis based on all first births in Bavaria (2015-18) provided by the Bavarian Quality Assurance Institute for Medical Care. Information on the mother and on the hospital were included. The Bavarian Index of Multiple Deprivation 2010 was used to account for area-level socioeconomic differences. Multiple logistic regression models were used to estimate the strength of association of the predicting factors and to adjust for confounding.

**Results:**

We included 195,087 births. Distances to perinatal centers were longer than to other hospitals (16 km vs. 12 km). 10% of women with documented risk pregnancies did not deliver in a perinatal center. Regressions showed that higher age (OR 1.03; 1.02–1.03 95%-CI) and risk pregnancy (OR 1.44; 1.41–1.47 95%-CI) were associated with choosing a perinatal center. The distances travelled show high regional variation with a strong urban-rural divide.

**Conclusion:**

In a health system with free choice of hospitals, many women chose a hospital close to home and/or according to their risks. However, this is not the case for 10% of mothers, a group that would benefit from more coordinated care.

## Introduction

Choosing a maternity hospital to deliver a baby represents an important decision in pregnancy for soon-to-be parents. From both the health system and the patient’s perspective, the choice of a maternity hospital should be based on individual needs and accessibility. Thus, the optimal maternity hospital is close to home and is adequate for the individual risk profile.

In the context of the German health system, there is a free choice of favored maternity hospitals [1]. This choice is complex and is made based on personal preferences such as that the hospital is already known, has been recommended by relatives or acquaintances, has a good reputation, or has high accessibility [2], and is known for elective hospital stays for several reasons such as for surgeries [2, 3] or oncology treatments [4].

The equipment and personnel present at a maternity ward differ between hospitals and are characterized according to the German hospital plan into perinatal centers (level I or II), perinatal foci, full-time maternity hospitals (with resident specialists in Obstetrics and Gynecology (ob/gyn)), and affiliated hospitals (with at least 500 births p.a. and less than 500 births p.a., no resident ob/gyn) [5]. They differ in the level, quality and quantity of care providedfor example, perinatal center level I has to have a 24 h presence of physicians and an additional on-call physician specializing in perinatal medicine, around-the-clock midwives, and a neonatal (intensive) care unit, as well as specific equipment [6]. Perinatal centers level I and II correspond to tertiary care providers in an international context. The Quality Assurance Guideline for Preterm and Mature Births of the Federal Joint Committee specifies that pregnant women with specific risk factors are recommended to deliver their children in a better-equipped maternity hospital (primarily a perinatal center) [6]. In optimized care, it is assumed that the health system provides optimal care for everyone.

Although several studies have shown that access and quality of care are relevant factors for hospital choice [2, 7], those studies rarely included patient risk factors. Scientific publications are scarce, especially with regard to the choice of maternity hospitals based on risk and/or accessibility,. In a review from the UK on how mothers choose maternity hospitals, pain care, continuous midwifery care, general atmosphere, and distance or travel time could be identified as factors [8], and urban or rural living environments play a crucial role [9–11]. In a Swiss study, professional competence and atmosphere were rated as top factors, along with proximity to the home [12]. Timely access to care measured in travel time to the point-of-care is a known indicator of accessibility to medical care, and can have an impact on health outcomes, especially in very rural areas [10, 13–15]. However, based on the literature, the choice of maternity hospital is motivated by similar factors identified in studies on other procedures: distance to the hospital or other healthcare providers plays a role, although it is rarely the first priority [12, 16–18]. Arguably, not everyone benefits from regulated optimized care, where mothers can choose with their health care providers the level of care that best meets their needs for the delivery of their baby, essentially indicating a trade-off between risk avoidance and proximity. Therefore, this study aims to identify the factors that influence the choice of maternity hospitals, focusing on the level of care provided by the hospitals, the (medical) need, and the distance the mothers would have to travel.

## Methods

This study is a cross-sectional observational study based on routinely collected data for quality assurance in the hospital sector in Bavaria, Germany.

### Data source

The data included all births in Bavaria from 2015 to 2018, documented by the Bavarian Quality Assurance Institute for Medical Care^1^. Bavaria is the largest of the 16 federal states in Germany in size and the second largest in population, including approx. 13 mil inhabitants (15.8% of the total German population as of 2018) [19]. The data is collected for the Federal regulated quality assurance and is published in regular quality reports [5, 20] and contains anonymized information on maternal characteristics and perinatal outcomes for all births in Bavarian hospitals. The dataset includes maternal information and the perinatal outcomes (e.g. Apgar score, birth weight, stillbirths, mode of delivery). The dataset was previously used for secondary data analyses [21–24].

The dataset was made available for this study in anonymous form for the years 2015-18. The Ethics Committee of the Medical Faculty of the LMU Munich issued a waiver for this study (23–0157 KB).

### Variables included

#### Information related to health need

We included only primiparous women in our study, assuming that previous birth experiences in one specific hospital influences a mother’s choice for the next birth. To assume a common level of knowledge and experience, we excluded all births to mothers with a birth history, which might reduce the representativeness of all births in our results but make the results more comparable within the group.

For the maternal information, we included age, body mass index (BMI), information on antenatal check-ups, and information on pregnancy risk. Age was defined as age at the date of delivery, and BMI was calculated at the beginning of pregnancy as the weight (in kilograms) divided by the square of the height (in centimeters). In Germany, risk pregnancies are first documented by the attending non-hospital resident gynecologist based on a risk assessment including numerous variables, such as maternal age, previous (failed) pregnancies, psychosocial concerns, or medical conditions. The attending ob/gyn at the hospital maternity ward then decides based on this information and other factors if this delivery is considered as a risk pregnancy.

#### Information related to level-of-care of the hospital

The level of care is available for maternity hospitals. It was categorized in levels L1 to L6 [5], with L1 representing the highest level of care to L6 with the least provision of care. For the analysis, we dichotomized this variable as perinatal centers (L1, L2) as tertiary care providers vs. non-perinatal centers. The outcome variable was therefore defined as “Chose a perinatal center” (yes/no).

#### Information related to living situation

The degree of urbanization was included as a three-category variable (“Cities”, “Towns and suburbs”, “Rural areas”) according to the EUROSTAT Degree of urbanization (DEGURBA)^2^ categorization, provided by the List of Municipalities Information System (GV-ISys) by the German Federal Statistics office^3^.

To calculate distances, hospital addresses were geocoded. Due to data protection reasons, mothers’ information on residence was reduced to a full zip code. We therefore approximated the starting point of the distance calculation, that is, the. mothers’ residence, using the geographic centroid of the zip code area. Distance is defined as shortest road distance in kilometers. We analyzed whether the selected hospital was the closest to the mother’s place of residence. If a more distant hospital was chosen, the level of care in this hospital was compared to that of the closest hospital.

To account for socioeconomic differences between the areas of residence of expectant mothers, we included the Bavarian Index of Multiple Deprivation for the year 2010 (BIMD 2010) [25, 26] in the analysis at municipality-level. The BIMD includes seven domains of deprivation (income, employment, education, municipal revenue, social capital, environment, and security) and was calculated for all municipalities. We categorized the municipalities by BIMD quintiles, with the first quintile (Q1) indicating the least deprived municipalities and the fifth quintile (Q5) indicating the most deprived municipalities. Zip codes for mothers’ places of residence were matched to the municipalities. If a zip code area had an overlap with two or more municipalities, this zip code was matched to the municipality with the largest area share, in line with literature [21–23].

### Statistical analyses

We calculated mean and standard deviation for continuous variables and absolute and relative frequencies for categorical variables.

We fitted multivariable logistic regression models to determine factors influencing the choice of a perinatal center (L1, L 2) as the place for delivery. The main factors of interest were the presence of a risk pregnancy and the travel distance. We adjusted for the number of antenatal check-ups, BMI at the start of pregnancy, BIMD, and degree of urbanity of municipalities. In the logistic regression analyses, we first analyzed a model including all deliveries for the outcome “Chose a perinatal center” (yes). We ran a fully adjusted model that included all variables of the mothers’ risk factors and potential confounders (area deprivation and urbanicity). We reported the exponential of the coefficients as odds ratio (OR). An OR > 1 indicates higher odds of choosing a perinatal center.

As special emphasis was placed on the group of mothers with a documented risk, an additional multivariable logistic regression model was formulated specifically for this subgroup. To account for the geographical hierarchy in the data, we also ran both analyses (full dataset and only risk-pregnancies) as multilevel models with a random intercept term for the mothers’ municipalities.

Distance measures were calculated using the routing option with the osrm package in R [27]. All analyses were performed with SAS (Vers. 9.4), and RStudio (Vers. 2022.12.0). Geographic analyses and maps were generated using QGIS (Vers. 3.22).

## Results

Data were available for 223,538 primiparous deliveries. Due to missing values in explanatory variables (BMI, *n* = 13,512, antenatal check-ups, *n* = 14,939) we included 195,087 women in the analysis.

Of all births, 53.1% occurred in perinatal centers (L1), and 7.3% in regional hospitals with less than 500 births per year (L6). Information on the mothers and the hospitals is shown in Table 1. On average, the women were 29.8 years old, had a BMI of 24.1 at the beginning of the pregnancy and had 12.0 antenatal appointment. 34.2% were documented risk pregnancies. Of all women, 27.5% lived in municipalities in the highest deprivation quintile (Q5) and 13.2% lived in the least deprived municipalities (Q1). Of all mothers, 51.8% chose the hospital closest to their home. Overall, the travel distance to perinatal centers I or II was slightly longer (16.0 km) than to the other hospitals (11.2 km).

**Table 1:** Cohort overview of all primiparous deliveries in Bavaria, 2015–2018

	N	%/mean	SD
**Age (years)**	195,087	29.84	5.07
**BMI**	195,087	24.07	5.77
**Number of antenatal checkups**	195,087	12.04	3.68
**Risk pregnancy**	67,831	34.77	
**Chose closest hospital**	99,612	51.06	
**Level of urbanization**			
Cities	73,688	37.77	
Towns and suburbs	66,400	34.04	
Rural areas	54,999	28.19	
**BIMD 2010 Quintiles (Q)**			
Q1 (least deprived)	25,743	13.20	
Q2	24,138	12.37	
Q3	25,985	13.32	
Q4	65,555	33.60	
Q5 (most deprived)	53,666	27.51	
**Classification of hospital**			
L1: Perinatal Center Level I	103,492	53.05	
L2: Perinatal Center Level II	22,352	11.46	
L3: Perinatal focus	28,999	14.86	
L4: Full-time obstetric clinic	15,766	8.08	
L5: Hospital > = 500 births p.a.	10,218	5.24	
L6: Hospital < 500 births p.a.	14,260	7.31	
**Distances (in km)**			
Distance (effectively travelled)	195,087	15.42	13.68
Distance (additionally travelled)	95,475	11.76	12.69
**Distance by hospital type**			
Perinatal Center I or II	125,844	16.04	14.85
Other hospital	14.3004	11.17	11.17

Of all mothers 37.8% lived in cities, 34% in towns or suburbs and 28.2% in rural areas. Mothers traveled an average of 15.4 km from their home to the hospital of their choice. Mothers who chose the closest hospital travelled an average distance of 9.1 km, while women who chose a more distant hospital (*n* = 95,475, 48.9%) travelled an average of 22.1 km. Taking into account the difference between the closest hospital and the chosen hospital, i.e. the difference between the actual distance and the distance to the closest hospital (see Table 1), women who chose a more distant hospital travelled an additional average distance of 11.8 km. Of these, 56.2% chose a hospital with a higher level of care than to the nearest hospital, 23.4% chose a similar level of care, and 20.4% as hospital with a lower level of care.

**Fig. 1:**
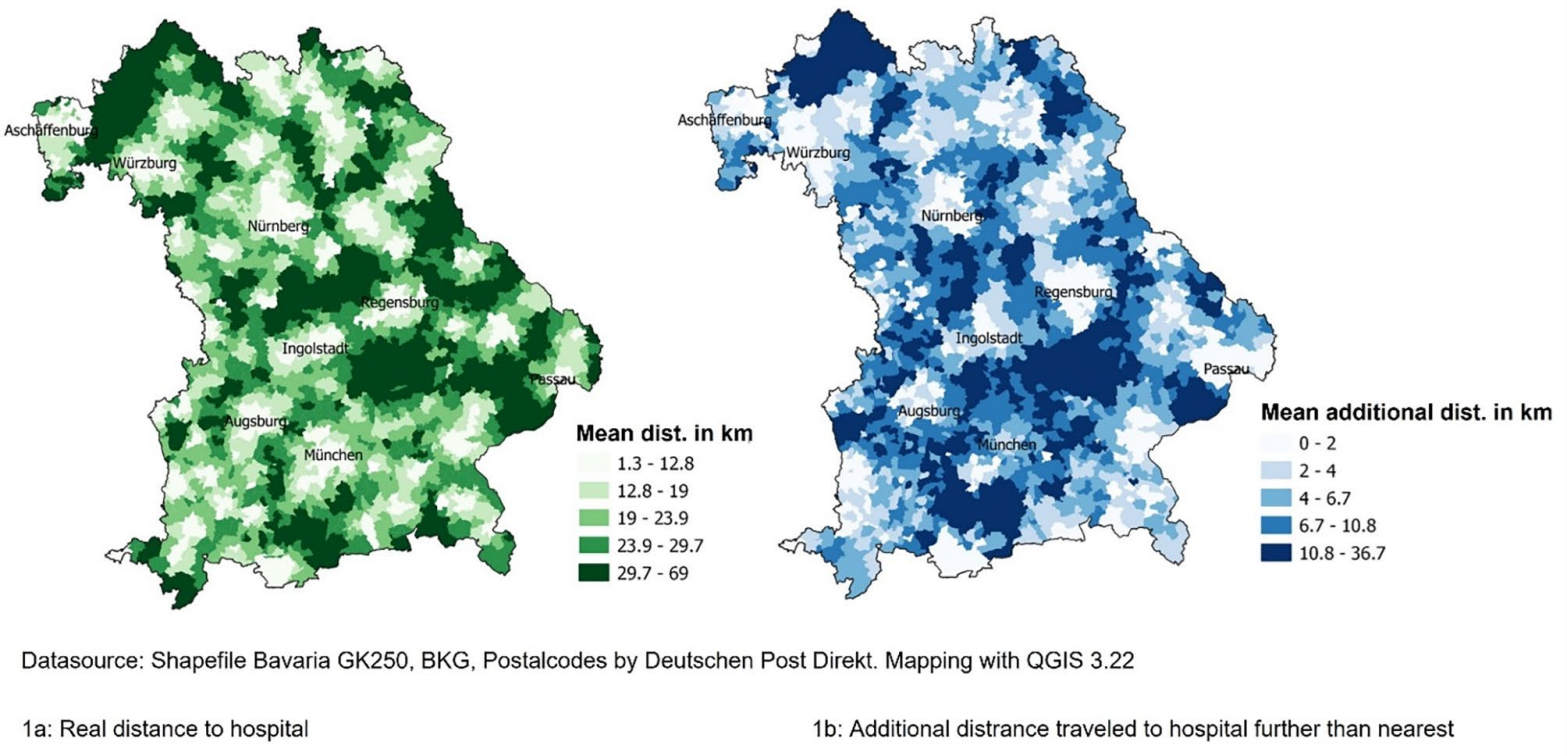
**a and b**: Distance travelled to the chosen hospital of birth (Fig a, *N* = 195,087) and additional distance travelled for a chosen hospital that was not the closest to the mothers’ home (Fig. b, *n* = 95,475)

To illustrate potential regional differences in the Bavaria, the real distances travelled are shown by aggregated zip code areas for all deliveries and the additional distances traveled for those who did not choose the closest hospital. Maps are shown in Fig. 1a and b.

The longest distances were travelled in the border regions in the north of Bavaria, and in the east (Lower Bavaria), and the shortest distances in the metropolitan regions of Munich, Nuremberg, and Augsburg (Fig. 1a). The cities are clearly visible as regions with shorter distances to the chosen hospital. If we consider the additional distance to travel to a more distant hospital (48.9% of all deliveries) mothers from rural regions traveled the greatest additional distance (Fig. 1b). Both distance maps (actual kilometers travelled and additional kilometers to travel to more distant hospitals) show a strong urban-rural divide.

**Table 2:** Bivariate information on documented risk pregnancies. We show absolute and relative frequencies as percentage for categorical variables and mean and standard deviation for continuous variables

	Risk pregnancy	
	no	yes
**Sample**	127,256 (65.23%)	67,831 (34.77%)
**Delivery in perinatal center I or II (N)**	77,763 (61.11%)	48,081 (70.88%)
**Chose the nearest hospital (yes)**	67,284 (52.87%)	32,328 (47.66%)
**Age (mean years)**	29.3 (SD 4.75)	30.83 (SD 5.50)
**BMI (mean)**	23.74 (SD 5.49)	24.68 (SD 6.22)
**Antenatal Check-ups (mean number)**	11.99 (SD 3.59)	12.15 (SD 3.84)
**Distance traveled (mean km)**	15.04 (SD 13.36)	16.15 (SD 14.24)
**Cities**	46,828 (36.8%)	26,860 (39.6%)
**Towns and Suburbs**	43,985 (34.56%)	22,415 (33.05%)
**Rural areas**	36,443 (28.64%)	18,556 (27.36%)

The results for documented risk pregnancies are shown in Table 2. Women with a documented risk pregnancy were more likely to give birth in a perinatal center (70.9% vs. 61.1%), and less likely to go to the nearest hospital to their home (47.7% vs. 52.9%). Among all women living in urban areas, 36.5% were documented as having a risk-pregnancy, compared to 33.74 in rural areas. Overall, 89. 9% of all deliveries with a documented risk pregnancy occur in perinatal centers.

**Table 3:** Multivariable logistic regression models. Model A includes all primiparous births in Bavaria, 2015-18, Model b includes only documented risk pregnancies. Odd Ratios > 1 indicate a higher probability of a delivery in a Perinatal Center

Model a: Perinatal Center chosen = yes, all deliveries (*N* = 195,087)			Model b: Perinatal Center chosen = yes, risk pregnancies (*n* = 67,831)		
***Intercept (Est, SE)***	*-0.6674*	*0.04*	***Intercept (Est, SE)***	*-0.559*	*0.0701*
	Odds Ratio	95% CI		Odds Ratio	95% CI
**Risk pregnancy (yes)**	1.441	[1.41,1.473]			
**BMI**	0.998	[0.996,0.999]	**BMI**	0.997	[0.994,1]
**Distance (km)**	1.052	[1.051,1.053]	**Distance (km)**	1.061	[1.059,1.063]
**Age (years**	1.025	[1.023,1.027]	**Age (years**	1.023	[1.020,1.027]
**Antenatal check-ups (number)**	0.994	[0.992,0.997]	**Antenatal check-ups (number)**	0.996	[0.991,1.001]
**ref.=Cities**			**ref.=Cities**		
**Towns and Suburbs**	0.135	[0.131,0.139]	**Towns and Suburbs**	0.130	[0.123,0.137]
**Rural areas**	0.080	[0.077,0.082]	**Rural areas**	0.074	[0.070,0.079]
**BIMD 2010 (ref = Q1, least deprived)**			**BIMD 2010 (ref = Q1, least deprived)**		
**Q2**	0.803	[0.773,0.834]	**Q2**	0.827	[0.772,0.885]
**Q3**	0.824	[0.793,0.855]	**Q3**	0.765	[0.716,0.818]
**Q4**	0.586	[0.565,0.607]	**Q4**	0.535	[0.502,0.570]
**Q5**	0.920	[0.889,0.953]	**Q5**	0.698	[0.655,0.743]

A second model was stratified on mothers with a risk pregnancy (Table 3, Model b). For all mothers, the odds to deliver in a perinatal center increased with the distance traveled (by 0,05 for each additional traveled kilometer) and if a risk pregnancy was documented (by 0.44). Compared to living in cities, living in less urbanized towns and in rural areas strongly decreased the odds of delivering in a perinatal center. Also, compared to mothers living in the least deprived municipalities, mothers in municipalities of any higher degree of deprivation had a lesser chance to go to a perinatal center, however, there is no clear trend visible.

For women with documented risk pregnancies predictors of attending a Perinatal Centers had the same direction but a higher magnitude of effect.

## Discussion

In our analysis of over 195,000 primiparous births, we found that over half of the mothers chose to deliver their babies in the hospital closest to home. Of those who chose a more distant hospital, mothers covered an average of additional 11.8 km, with 21.6% of those choosing a maternity hospital with higher levels of services provided. Only 10% of mothers with documented risk pregnancies did not choose a perinatal center for delivery.

Only few studies have focused on travel distance and quality of care delivered at maternity hospitals in a high-income country setting. Our results align with the results of Avdic et al. [28], showing that mothers were willing to travel an additional 0.1 to 2.7 km to a hospital with higher quality. Their analysis assessed whether a pregnant woman had a hospital diagnosis indicating a risk pregnancy or was admitted as an emergency; however, any information on the pregnancy prior to hospital admission could not be included [28]. Another study set in an urban context analyzed by Zeitlin et al. [29] showed that women travelling longer distances and women living in more deprived neighborhoods were less likely to deliver their preterm babies in perinatal centers with the highest quality of care [29]. When considering travel distance for hospital choice generally, several articles showed that distance is the main driver for choosing a hospital for elective visits, showing that even when quality is rated an important selection criterion, a close-to-home hospital is more likely to be chosen [9, 10, 30]. Also, other studies have shown an association between accessibility of emergency care and factors such as the quality of the hospital, individual socio-demographic status, or rurality [13, 31].

Several studies role of (or lack thereof) from Low-and Middle-Income Countries, have shown that longer travel distances are associated with general access to hospital birth/skilled birth attendance or antenatal care [32, 33], or adverse outcomes such as fetal mortality [34–36]. Those studies also point out other influencing factors such as individual socioeconomic status [32, 37]. However, due to the different setting, and the rather high hospital density in Bavaria/Germany and financial coverage of services, the results are not easily comparable.

In our study, the majority of deliveries already take place in hospitals wihth a high level of care, many of which would probably be safe deliveries in hospitals with a lower level of care. However, since our results show that more than half of the deliveries occur in the hospital closest to the mothers’ home, this may be due to of convenience.

Area deprivation was associated with the likelihood to deliver in a perinatal center. Women living in areas with higher deprivation were less likely perinatal centers. This was independent of factors such as BMI or the degree of urbanization. This reflects the findings on birth outcomes from studies based on the same data source, showing that area deprivation was associated with, for example, preterm birth [23] or a lower detection rates of gestational diabetes [22]. The results showing an association between area deprivation and unfavorable outcomes should be analyzed in more detail, including primary data (especially on socio-economic information and factors influencing hospital choice), in order to identify possible policy solutions.

### Sensitivity analyses

We included area deprivation and the degree of urbanization of the municipality of the mothers home in a logistic regression model. Since the BIMD 2010 and the degree of urbanization were used at the municipality level and the variables on personal risk factors variables were based on the individual level of the mother, clustering effects could be overseen. We therefore also ran both models also as multilevel models, including a random intercept term for the mother’s municipality of residence. Since the dataset includes 1,852 municipalities, this added a higher variance but did not change the fixed effects of the variables. Only the magnitude of the effect for the most deprived municipalities and the effect for the degree of urbanization were weaker in the multilevel models, as expected when controlling for areas. Also, the logistic model showed a better fit without the random term. We therefore decided to report the non-hierarchical model.

### Strengths and limitations

This analysis is a secondary data analysis, the data was not collected for research purposes but for quality control purposes. Our analysis is based on all primiparous births in Bavarian hospitals regardless of the mother’s health or socio-economic status, language skills, or place of residence, which minimizes selection bias to a minimum: Only home births and births in midwife-led units could not be included, which account for approx. 1.5% of all births^4^. However, several limitations of the dataset and methods need to be mentioned. In addition, although the study covers almost all births, the study results are only valid for the years 2015–2018 and only for the Federal State of Bavaria, which limits the generalizability to a German or international population.

The distance from the mother’s home to the hospital was calculated using the mother’s full zip code. This was an approximation that is less accurate for small differences in distance, but probably sufficiently reliable for larger distances. With this in mind, we are likely to misinterpret the “nearest hospital” in urban areas, where the differences in distance between the closest and second closest hospitals are smaller and may therefore depend more on the approximation of the centroid of the postal code. However, we assume that this error is random and that it also occurs more often for selected perinatal centers (since they are more clustered in urban areas). As shown in Table 1, the additional distance traveled is 11.76 km - if we only consider the additional distance to the second closest hospital, where the postcode approximation error is most likely to occur, the average additional distance is still 5.72 km. With these considerations in mind, we expect the impact of the variation in distance due to zip approximation on our results to be minimal. We included only first births in the analysis to assume a common level of knowledge. For follow-up research projects, including all births may provide a broader understanding of the factors influencing maternity hospital choice.

We could also not include individual information on socio-economic status. We used the BIMD 2010 as a measure of area-level deprivation, serving as a proxy for the differences in living conditions. However, including the individual socio-economic status and information about health literacy or health behavior could lead to different results. The BIMD is mostly based on data from the 2010reference year. However, area deprivation tends to change slowly, and since we used aggregated area deprivation quintiles, it is unlikely that major changes occurred in the years between 2010 and our 2015-18 data.

Although we were able to analyze a comprehensive dataset, awareness of the distinction between hospital levels of care, as well as the health literacy of pregnant women, their families, and also communication with their ambulatory care providers, may have changed in the years since 2015-18. For example, the Institute for Quality Assurance and Transparency in the Healthcare System’s (IQTiG) perinatal center information website^5^ was launched in 2014, so it was likely not well known in our first data year. Other data sources, such as hospital quality reports, as well as an ongoing public health policy discussion about possible minimum case volumes, will have raised awareness and may have already influenced the results we showed in our analysis.Our main limitation is that while it may be desirable from a health system perspective that women choose their birth hospital solely on the basis of accessibility, potential risk, and level-of-care, many other factors play a role in this decision. Although many potential determinants of maternity hospital choice are not captured in the perinatal data (e.g., recommendations from acquaintances and physicians, preferences for delivery room facilities), our dataset can provide insights into care patterns and distance travelled. Further analyses should be conducted with additional predictors such as the mothers’ individual opinions to address the decision-making process, important factors identified in other studies [18, 38, 39].

## Conclusion

In our dataset, only 10.1% of all mothers with a risk pregnancy did not choose a perinatal center for delivery. These women lived in more deprived areas, had a higher BMI, lived in more rural municipalities and were slightly older than average. From a health policy perspective, the lack of navigation assistance in a free-choice system could be to a problem for this specific group and requires further evaluation. Helping expecting mothers to make an informed decision about accessibility and quality of care is urgently needed in a healthcare system where they can choose the hospital freely and patient navigation may be lacking. The results should be taken into account for local hospital planning from both a processing and a system side to potentially reach those 10% of women who did not deliver their child in accordance with their personal risk. Also, the attending gynecologists outside as well as within hospitals could serve as partners for an open discussion about the hospital choices. Regarding closing smaller hospitals with lower levels of care, opening potentially new hospitals, or bundling of specialized care, it is relevant that mothers who need higher-level-of care are able to access and ultimately choose that care. The results show that many expecting mothers do already choose a hospital (a) based on their potential risks or (b) a high-level-of care hospital that is close to their home. In contrast, pregnant women who do not fall into these categories need to be the focus for more transparent information and guidance in the health system.

## Footnotes

1 As of January 1st, 2022 the Bavarian Quality Assurance Institute for Medical Care (BAQ) fused into the LAG Bayern, now additionally responsible for the quality assurance of medical care delivered by primary and specialists care outside of hospitals.

2 https://ec.europa.eu/eurostat/web/degree-of-urbanisation/background.

3 https://www.destatis.de/EN/Themes/Countries-Regions/Regional-Statistics/_node.html.

4 There is no clear documentation for non-hospital-based births. The German Society for quality in out-of-hospital obstetrics approximates the numbers by calculating the difference of the total number of births and the quality-controlled number of hospital births, see: https://www.quag.de/quag/geburtenzahlen.htm (in German).

5 perinatalzentren.org, in German.
